# Vaccination against SARS-CoV-2: a human enhancement story

**DOI:** 10.1186/s41231-021-00104-2

**Published:** 2021-12-04

**Authors:** Niklas Alexander Döbler, Claus-Christian Carbon

**Affiliations:** 1 Department of General Psychology and Methodology, University of Bamberg, Bamberg, Germany; 2 Research Group EPÆG (Ergonomics, Psychological Aesthetics, Gestalt), Bamberg, Germany; 3 Bamberg Graduate School of Affective and Cognitive Sciences (BaGrACS), University of Bamberg, Bamberg, Germany

**Keywords:** Vaccination, Human enhancement, Ethics, Psychology, Public Health, Policy making, Politics, Pandemic

## Abstract

**Background:**

Vaccination is an essential strategy for mitigating the COVID-19 pandemic. Besides its significance as a public health measure, vaccination is a sophisticated example of modern biotechnology. Since vaccination gives the human body an ability that it does not naturally possess, the question arises as to its classification as Human Enhancement.

**Main Body:**

Exemplified on a selection of different definitions, we conclude that vaccinations may indeed be classified and treated as a form of Human Enhancement. This raises some ethical issues that are notorious in the broad field of Human Enhancement. A study with *N* = 67 participants revealed that vaccinations are perceived neither as a clear nor poor example of Human Enhancement.

**Conclusion:**

We argue that qualifying vaccination technology as Human Enhancement does not provide convincing arguments to reject vaccination. By examining the Human Enhancement debate and the similarities to the issue of vaccination shown here, policymakers can learn valuable lessons regarding mass vaccination programs’ current and future handling.

## Introduction

Vaccination against SARS-CoV-2, if scientifically tested for effectiveness, approved by respective health authorities, and regularly administered, is a promising tool for mitigating this pandemic and future epidemics [1, 2]. Besides reflecting vaccination as a global and public health issue, the mere application of this technology tackles a series of psychologically relevant themes, starting with simply being afraid of needles [3], to getting an injection of an unknown, not well understood active agent [4], even as far as interpreting vaccination as ill-intended campaigns framed within conspiracy theories [5]. Despite these often debated and societally significant issues, we will focus here on a different perspective on vaccination which is largely neglected in the public debate: Vaccination can also be interpreted as a powerful means of *Human Enhancement*.

There are several definitions of Human Enhancement, but in a fairly broad conception, it is seen as creating better people by means of technology [6, 7]. When more specific definitions are examined, it will be concluded that any vaccine against SARS-CoV-2 qualifies as a Human Enhancement. This categorization comes along with significant ethical implications. Some critics of Human Enhancement are fearful of the idea of the widespread application of it and fear that it will threaten the nature of humanity [8, 9]. Yet, in order to be effective, any vaccine against this pandemic disease must be administered to as many people as possible. So, will ending this pandemic lead us to a dark path, leaving the essence of being human behind us?

### Definition of enhancement

Although the debate about Human Enhancement is carried out with great passion, the term has no standard definition. Table 1 features a selection of definitions. It becomes evident that all of these approaches differ in important aspects like the means of enhancement and the desired end state. Whilst Buchanan [10], Coeckelbergh [7], and the President’s Council on Bioethics [8] explicitly mention science and its application by technology, in particular, Daniels [11], Allhoff et al. [12], and Juengst [13] do not limit their definitions to specific means of application. Another demarcation point is the desired outcome and its realization. The question that arises here is whether the intervention must be successful to be considered Human Enhancement or whether the mere goal of improvement is sufficient. The general terms “better,” “improvement,” or “enhancement” always refer to an end state, not realized yet, which is always contrasted against the undesired status quo. Noteworthy is that the end state is often not fully defined except for the vague implicit reference of improvement.

**Table 1:** Different definitions of Human Enhancement and their components

Source	Definition	Specifies means of application	Success criterion	Specific enhancement target	Beyond a specific/normal range	Humans as application subject
Allhoff et al. [12]	Strictly speaking, “human enhancement” includes any activity by which we improve our bodies, minds, or abilities—things we do to enhance our well-being.		x	x		x
Buchanan [10]	A biomedical enhancement is a deliberate intervention, applying biomedical science, which aims to improve capacity that most or all normal human beings typically have, or to create a new capacity, by acting directly on the body or brain.	x		x	x	x
Coeckelbergh [6]	Human enhancement aims at using technology to create better humans.	x				x
Coeckelbergh [7]	Human enhancement can be defined as the improvement of humans by technological means.	x	x			x
Daniels [11]	The treatment-enhancement distinction draws a line between services or interventions meant to prevent or cure (or otherwise ameliorate) conditions that we view as diseases or disabilities and interventions that improve a condition that we view as a normal function or feature of members of our species.		x	x	x	x
Juengst [13]	The term enhancement is usually used in bioethics to characterize interventions designed to improve human form or functioning beyond what is necessary to sustain or restore good health.			x	x	x
President’s Council on Bioethics [8]	“Enhancement,” by contrast, is the directed use of biotechnical power to alter, by direct intervention, not disease processes but the “normal” workings of the human body and psyche, to augment or improve their native capacities and performances.	x	x	x	x	x

*Note.* Defintions are presented in alphabetical order of the authors

Which capacity must be enhanced, and when is any improvement so significant that the intervention in question qualifies as Human Enhancement? Juengst [13] mentions “good health” as a reference value, but this is a flexible threshold [14]. Buchanan [10], Daniels [11], and in some sense, the President’s Council on Bioethics [8] suggest the normal abilities of a prototypical human being as the reference frame, while Buchanan [10] does not specify any specific threshold inside this functioning. However, the “normal functioning” approach is highly flawed due to the inability to clearly identify the defining features of what it means to be “human” or “normal” [15–17]. Lastly, Coeckelbergh [6, 7] does not mention any definitory restrictions regarding outcome parameters or a benchmark. Allhoff et al. [12] are very non-specific in their mention of general improvement in abilities or overall well-being. However, besides the quest for improvement, every definition shares one aspect: Any enhancement, regardless of the reference frame or success, must happen *to* someone, to a particular individual. Human Enhancement requires a person to be enhanced and, as such, this phenomenon encompasses an extensive range of possibilities, methods, techniques, and technologies.

### Vaccination as technology

Before going into detail about whether or not vaccination reflects a form of Human Enhancement, its classification as technology must be discussed. Even if the term “technology” is not distinctly mentioned in some of the aforementioned definitions, it is clear that ethical concerns directed at enhancement interventions may be based on the applied means rather the desired outcome [13, 18]. The Human Enhancement debate revolves around the question of how far we may go beyond humans’ natural development and adaptation when facing specific challenges. Such challenges might be general physical or intellectual barriers, such as overcoming natural limits of our bodily capabilities or mental capacities. Challenges can exist regarding natural developments of our body and mind due to senescence but can also emerge due to natural or human-made changes to the environment [19–21]. Here Human Enhancement can be seen as a matter of control over natural processes or conditions, which are experienced as undesirable and flawed [19]. The means by which this control over nature is commonly exercised is technology [7, 22–24].

But what is technology? Carroll [22] carried out a careful and comprehensive ethological analysis of the timeless essence of technology. She argued that any technology consists of three core aspects: the possession or ascription of a *function* (I.) related to a specifically designed or afterward identified *purpose* (II.) that will eventually lead to some sort of *benefit* (III.). Another important aspect is the ability to comprehend and appreciate the notion of technology, described as reflexivity. Accordingly, this ability is contingent on and carried out through these essential features [22]. Technology – according to Carroll – comprises anything identifiable by humans as having a specific function to fulfill a certain purpose that will lead to a beneficial outcome, whether or not material or simply knowledge-based. In other words, if it can be used to create a favorable outcome, it is technology, regardless of its origin and physical status. While the latter might sound controversial, it is essential to note that Carroll [22] emphasizes the role of creation and organization in the emergence of technology. However, she does claim that it is irrelevant if human beings themselves created this organization. The only uniquely human element is in the identification of the function and purpose.

A discussion about the general usability of this admittedly abstract definition is outside the scope of this paper. Nethertheless, Carroll’s approach offers a fascinating perspective for the assessment of the status of vaccination regarding Human Enhancement: Depending on the type of this health intervention, different natural occurring entities and processes are exploited and sometimes deliberately altered to provoke an immune reaction [25, 26] to achieve the beneficial outcome of a resistant organism and, in the long run, to successfully mitigate epidemics threatening the health, wealth and peace of entire human societies. Hence, the creational process of identifying the relevant mechanism within the functional biological interplay of pathogen and immune response, exploiting and provoking it in order to fulfill the intended purpose, qualifies any application of vaccination as technology. In this sense, vaccinations are also technologies in the notion of Allenby and Sarewitz [23]: “cause-and-effect machines, linking a human intent to a particular consequence via the embedded function of the technology” (p. 36), with the function being the creation of individual immune protection.

### Vaccination as human enhancement

Several scholars [10–12, 23, 27–29] have discussed or briefly mentioned vaccination as a kind of enhancement, but we will discuss this issue in more detail. In general, any effective vaccination aims to improve human capability, viz. the human immune system. The purpose of enhancing a human capacity is the defining feature of any Human Enhancement technology. Concretely, the application of vaccination technology is about human adaptation to an environment hazardous to health. Following this line of argument, this realizes the ideal of being a cyborg, who, according to Clynes and Kline [30], “deliberately incorporates exogenous components extending the self-regulatory control function of the organism in order to adapt it to new environments” (p. 27).

By administering a vaccination, we aim to strengthen our immune system, making it better than it was. This intervention is carried out through bio-technology, directly acting on the human body and its species-typical functioning. The latter part is important. If the human immune system responded adequately to the COVID-19 causing SARS-CoV-2 infection, there would be no need for enhancement. Hence the “normal functioning” allows no conclusion about the adaptiveness of the same function in the face of different environmental threats. The human immune system may work completely inside its normal parameters but may be unable to neutralize a pathogen. There are, furthermore, serious difficulties in determining the “normal” or the “human” [15, 17]. Immune reactions to SARS-CoV-2 display a significant variability as severe medical conditions resulting from this disease depend heavily on age and pre-existing conditions [31, 32]. This means that the ability to mitigate the threat from this particular virus is not sufficiently distributed to eliminate its danger. That is, humans’ normal capability to repel SARS-CoV-2 pathogens is in desperate need of enhancement.

This improvement is realized by technology, here bio-technology. Vaccination teaches the human immune system about specific characteristics of the pathogenic agent. This strategy does build on already known mechanisms of the body and improves it by adding new capacities. In their approach, Allhoff et al. [12] differentiate enhancement technologies based on an internal-external distinction. Accordingly, only technology integrated inside our bodies would qualify as an enhancement. Needless to say, any vaccination must be applied inside the human body in some way, typically intra-muscularly, nasally, or by oral application.

There is a comprehensive debate whether or not a treatment-enhancement distinction is suitable for the Human Enhancement issue [8, 11–13, 16, 27, 33]. Due to the presence of a deadly virus and a resulting pandemic, one could prematurely classify any vaccination against SARS-CoV-2 as a treatment. However, in order to cure or treat an adverse condition, this condition has to be present. Juengst [13] noted that any preventive measure is used with the same disease controlling intention as a treatment. Certainly, vaccination against SARS-CoV-2 is intended to control a disease, not only on the individual but also on a societal level. But ultimately importantly, this enhancement strategy is done a priori to a *potential* infection but not as a response to an *actual* infection. Thus, vaccination should not be categorized as an immediate treatment but as a general enhancement for the unfortunate case of needing such enhanced capabilities.

Some authors make a distinction between “historical/conventional/natural” and “biomedical/unconventional” forms of Human Enhancement [7, 10, 12, 28]. The former may prima facie qualify as Human Enhancement, but its means are so well established that its use and dissemination does not raise major ethical concerns. Buchanan [10] names agriculture and literacy as examples, while Allhoff et al. [12] add a healthy diet to their definition of “natural” enhancements. With the first extensive vaccination campaign dating back to the transition between the 18th and 19th centuries, this technology is a product of human societies’ increasing scientific and technological progress [34, 35]. But at what point does a technology cease to qualify as “unconventional” and changes to “conventional” or “natural”? Since conventional vaccination technology enables the human body itself to produce a successful immune response, this type of vaccination could easily be classified as natural, similar to strengthening one’s immune system by having a balanced diet, getting more sleep, or eating vitamin-rich fruit. But such simplification would neglect the important public debate on people who are scared or at least concerned or skeptical about becoming vaccinated because they fear a variety of possible negative consequences [36–39]. So, even “conventional” enhancements may be controversial. Yet, this does not disqualify them from being an enhancement.

With such conflicts in differently assessing enhancement in general, we have to conduct thorough case-by-case examinations of respective technologies in order to be able to qualify whether a concrete enhancement method should be applied or not. For example, swords certainly enhance one’s ability to fight against an offender and are among the oldest pieces of technology human civilization has produced. Yet, most societies agree that swords should be regulated in some way. Thus, the question of whether a technology is a conventional or an ethically problematic extension of human capabilities urgently requires clarification by both proponents and critics of enhancements [7].

Given the explicit and implicit notion of vaccination as Human Enhancement, it is not surprising that some aspects of the ethical debate about these phenomena are similar. There are some concerns about the gene-changing potential of the new and revolutionary mRNA technology. Implementation depth, here on a genetic level, may be a source of intuitive ethical unease towards some enhancement technologies. This unease holds, even if the transformative potential does not differ among different technologies and their respective depth of implementation [18, 40, 41]. Reservations against a SARS-CoV-2 vaccination [5, 42, 43] may result from this general discomfort and the fact that vaccinations are a highly complex technology with a special relation to what is called “transparency” by some techno-philosophers. On the one hand, this attribute is used to describe artifacts whose functions are easily understandable and accessible; things that can be functionally restored [44]. Under this notion, vaccines are non-transparent and may fulfill the warning of Human Enhancement and cloning critic Leon Kass [9]:

*In contrast, biomedical interventions act directly on the human body and mind to bring about their effects on a subject who is not merely passive but who plays no role at all. He can at best feel their effects without understanding their meaning in human terms (p. 22).*

On the other hand, vaccinations are nearly *totally transparent* [45] in the sense that they apply their function in such close interaction with the body that the technology and the human become virtually inseparable. Either way, the human-technology relation is altered, and this can result in ethical concerns. While the academic literature already explicitly considers vaccination as Human Enhancement, the ethical unease of people who oppose vaccination may be explained due to a similar subconscious classification. In this sense, rejecting vaccination because it changes the body in an unacceptable manner or beyond a “natural” threshold acknowledges the status of this technology as Human Enhancement. Opposition to vaccination and Human Enhancement for this reason, therefore, may be two sides of the same coin.

This was impressively shown by recent news coverage of the German protest group “Querdenker” (“Lateral Thinkers”), who oppose not only public health measures but also reject vaccination against SARS-CoV-2 [46]. It was clearly evident that this group’s arguments in their opposition to vaccination resembled familiar arguments in the general Human Enhancement debate. Protagonists stated that their rejection of vaccinations was grounded on their notion of a fundamental conflict between science and nature. They rejected the implicit notion that the human immune system was insufficient to deal with a natural threat and thus must be enhanced. Demonstrators feared that human nature was at stake. Other concerns were expressed regarding an alleged “transhumanist agenda” and the dangers of genetic monocultures. A survey of this protest movement’s political attitudes showed that its supporters were largely convinced about the potential of our natural healing powers and a supposed divergance from nature [47]. Not only the metaphor of a monoculture but also the danger of transhumanism and the need for the preservation of human nature are arguments common to so-called *bioconservatives* [9, 48–50]. This group opposes Human Enhancement for various reasons beyond the scope of this paper. However, these ideological movements’ superficial resemblance is an important observation and should be focused on in future research [51].

Overall, new and revolutionary vaccination technologies are bearing the brunt of a fair amount of uncertainty. It is up to scientists and scientific journalism to communicate the function, benefits, and limitations of these Human Enhancement technologies. Otherwise, frustration due to unrealistic expectations may hinder the global effort in fighting the pandemic. The same is true for Human Enhancement technologies in general. Here, expectations about their transformative potential may be misled, resulting in unsubstantiated fears, hopes, and deadlocked debates [52–55]. Another parallel can be identified in the concerns of being one of the first individuals new vaccinations are administered to [42]. In the case of cognitive enhancement, individuals were more likely to affirm its use by others than by themselves [56].

The question of how any enhancement technology will be distributed is a recurring topic within the debate [57–59]. The equivalent in the vaccination debate is the question of whether immunized individuals should be granted more privileges than those that are not. Any answer to this question must address the fair distribution of vaccinations. The idea and implementation of immunity passports, either by vaccination or having had the illness, may exacerbate the existing socio-economic struggles among populations [60, 61]. Due to its qualification as an instance of Human Enhancement, lessons learned from the question of immune privileges will help evaluate future and current enhancement technologies.

The positive sides of getting a vaccination are manifold. It is crucial not only to consider the direct effect on the individual human immune system but also proximal outcomes of various kinds [23]. Here, herd immunity or fewer infections, in general, may allow unvaccinated individuals to benefit from the widespread administration of SARS-CoV-2 vaccination as a so-called *free rider*. Strictly speaking, this *enhancement-by-others* or “network effect” [10] does not count as Human Enhancement, as there is no direct link between the enhancement technology and the free-riding individual. Yet, this emphasizes the importance of recognizing the different levels of effects of bio-technology and Human Enhancement. Under the notion of restored freedoms for vaccinated people, one could consider vaccinations as a “positional good,” meaning that its benefit results from unequal distribution and the fact of other people *not* having this enhancement [27]. However, as Buchanan [10] has discussed, due to the concept of herd immunity, vaccinations may result in one of the aforementioned “network-effects,” meaning that the more people are enhanced, the greater the positive outcome. If vaccination ends the pandemic, the application of technology to a majority of people has resulted in collective and group-related beneficial outcomes. But it is essential to recognize that any positive societal or economic effect due to mass vaccination is constituted by a bottom-up process, i.e., as many vaccinated individuals as possible. Hence, it is essential to consider the long-term perspective and moderate the restoration of freedoms to minimize social tensions among vaccinated and non-vaccinated people and simultaneously foster the emergence of network effects, i.e., by ensuring a high vaccination rate all around the globe.

As COVID-19 has severe negative impacts on social-economic parameters [62], interventions against it – whether immediate treatment inside an intensive care unit or preventive enhancements – must be evaluated within the same dimensions. In the middle of a global health crisis, a second social crisis emerges alongside it. Vaccination can be a fruitful intervention here, yet besides its beneficial effects on individual health, it would also be a medical solution against social problems. This type of approach was warned against by Juengst [13], who rejected the idea that biomedicine should intervene inside the realm of social policy. While Juengst [13] comprehensively discusses his approach’s limitations, it becomes evident that medical issues and their accompanying social equivalents within a global pandemic are inseparable. Thus, vaccinations against SARS-CoV-2 are not only a medical enhancement but also a social one. See Table 2 for an empirical study addressing the research question whether vaccination is perceived as Human Enhancement.

**Table 2:** Study: Are Vaccination Perceived as Human Enhancement?

**Procedure** To explore whether vaccinations are seen as Human Enhancement, an online survey was conducted. Participants were told that Human Enhancements are special technologies but that this term has no standard definition. They were then introduced to Coeckelbergh’s [7] definition as a possible example of a definition. We chose this definition due to its relative broadness. Participants were then asked to rate fifteen different technologies on a Likert-scale, whether or not they viewed them as 1 *– No example* to *7 – Clear example* of Human Enhancement. Afterward, they had the opportunity to fill a free-text question, answering what types of Human Enhancement they were already using. Participants were then asked to state their attitude to a variety of human practices, i.e., genetic engineering of plants, animals, or humans, vaccinations, Human Enhancement, human interference with nature (dams, mining, etc.), and space colonization (Likert-scale, *1 – Strong rejection* to *7 – Strong approval*). We limited our analysis to the attitude on Human Enhancement and vaccinations. We intend to use the data for further analysis and share them on OSF. Lastly, participants answered if they were already vaccinated against SARS-CoV-2 or were willing to do so and are generally concerned about keeping their vaccination status against other pathogens updated. The Survey language was German, and university students could apply for course credit.	
**Participants** Only participants who complete the whole survey were included. One participant was excluded due to a self-reported lack of seriousness in completing the survey. This led to *N* = 67 (40 M, 26 F, 1 D). Mean age was 31.67 years (*SD* = 11.51, range 17-60 years). The sample was mostly educated, with 84% (*n* = 56) at least holding a High-School degree. 97% (*n* = 65) reported having already received vaccination against SARS-CoV-2 or be willing to do so. 73% (*n* = 49) reported being generally concerned about keeping their vaccination status updated.	
**Results** Mean ratings on whether the example was considered an example of Human Enhancement are shown in Fig. 1. Concerning their perception as Human Enhancement, vaccinations were evaluated neither as a clear, nor as a poor example (*M* = 3.79, *SD* = 1.90, range = 1-7, *Md* = 4). Their rating was the eighth highest. The rating of vaccinations as an example of Human Enhancement did not correlate significantly with the general attitude on vaccinations (*M* = 6.52, *SD* = 0.82, range = 3-7, *Md* = 7) *r* = 0.22, *p* = 0.08, but positively with the attitude on Human Enhancement (*M* = 4.78, *SD* = 1.51, range = 1-7, *Md* = 5) *r* = 0.34, *p* = 0.004. Attitudes on Human Enhancement and Vaccinations did not correlate significantly *r* = 0.22, *p* = 0.08.	
**Discussion** The results presented here support the notion that vaccinations are perceived in part by the public as a form of Human Enhancement. Yet, they are not viewed as much as prototypically cybernetic prostheses or cochlear implants. Out of the answers to the free text question on which examples of Human Enhancement participants already were using, only 3 out of 62 given examples mentioned vaccinations, even though this question was asked after the survey put vaccinations in a possible Human Enhancement context and the majority of participants stated a general engagement with at least some sorts of vaccinations (For comparison, “Glasses” were mentioned fifteen times). This suggests that even vaccinations are generally not perceived as Human Enhancement by people in their daily lives. Attitudes on Human Enhancement vaccinations, in general, did not correlate significantly. This may be because of the ethical complexity of these issues. Nevertheless, as we have shown, there are some similarities in the debate of these technologies. However, this does not mean that those who oppose vaccinations do so because they see it as a form of Human Enhancement. Yet, people who saw vaccinations as an example of Human Enhancement showed a more positive attitude to this phenomenon. This may be significant for promoting positive attitudes toward other/future forms of Human Enhancement, as this finding suggests that a widespread and useful example of Human Enhancement that is explicitly viewed as such may promote general attitudes toward these technologies. Our data provide a first empirical perspective on the public notion of vaccinations as Human Enhancement. The media extensively covered vaccination against SARS-CoV-2 and its beneficial effects in mitigating the pandemic by applying modern biotechnology. This contextual effect may have shaped the evaluation of vaccinations, making its functional status as a form of Human Enhancement more salient. Yet the overall rating and free texts answers suggest that even under these circumstances, vaccinations are not perceived as a prime example of Human Enhancement. The functional status of capability enhancement of vaccination technology remains ethically controversial. It is therefore important to conduct future research into this topic, especially after the current pandemic. Our sample mainly consisted of people willing to receive a vaccination shot against SARS-CoV-2 and showed a very positive attitude towards this topic. Future research must address a heterogeneous population and include people who are skeptical towards vaccination and Human Enhancement in general, especially considering the framing of vaccination as modern biotechnological enhancements.

**Fig. 1:**
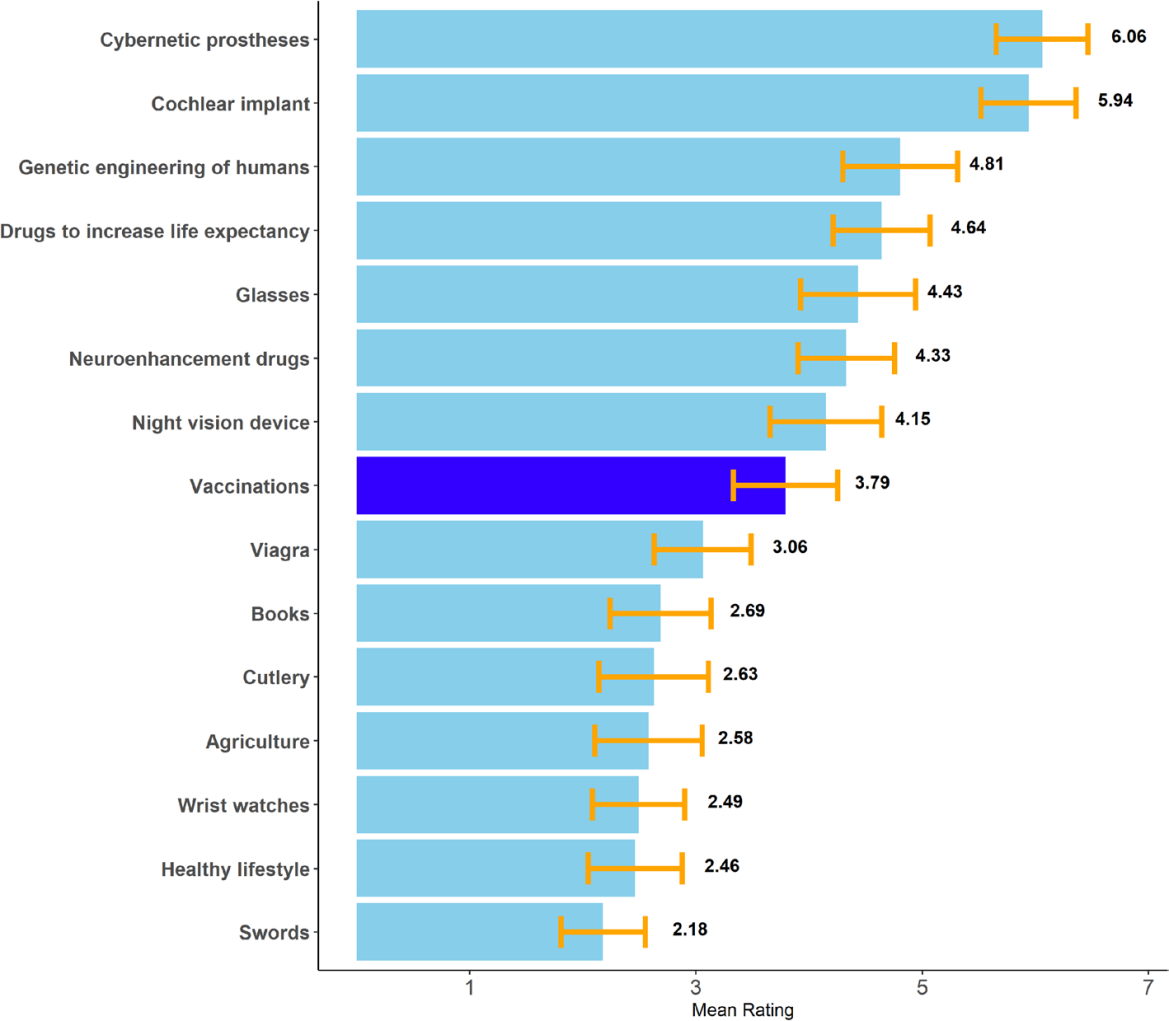
Ratings on the Prestented Examples. Orange bars represent 95%CIs

## Discussion

The current COVID-19 pandemic is a highly complex and dynamic situation. On a medical and individual level, the course of the disease may vary enormously. Some infected individuals will experience little to no symptoms [63], while others will suffer severely, including facing the possibility of death. Seen globally, infection prevention measures are causing social-economic, cultural, and societal disruptions worldwide. The answer to this threat includes the widespread application of biomedical technology in order to improve a human characteristic beyond what is necessary to maintain a “normal” healthy condition. In other words, the application of Human Enhancement technology seems to be mandatory.

In a medically and technologically advanced world, excluding Human Enhancement ideas from measures to mitigate a deadly pandemic is an ethical problem. The history of epidemiology shows that several pandemics with very different pandemic severity indices were all survived by humankind, even without vaccination technology available [64, 65]. Yet, because science possesses the capability to develop and successfully apply vaccines, this option should not be prematurely excluded just because it is a form of Human Enhancement. Many bad medical conditions can be prevented, lives can be saved, and pandemics can effectively be mitigated to be able to much sooner switch back to more normal states of everyday life. Within this approach, and always carefully ensuring that individuals’ safety and autonomy are guaranteed as good as possible, Human Enhancement via vaccination is justified.

As we have shown in various examples, definitions of Human Enhancement include vaccination technology. Depending on the individual attitude towards this issue, this may not be a problem. However, some voices fear the widespread application of Human Enhancement [8, 9]. Under this notion, the acute pandemic is bad news, as vaccination seems to be the most promising approach for ending this global health crisis. Here Mitrovíc [51] states that: “in the pandemic […] enhancement, otherwise ethically difficult to accept, may find its way to users (groups and individuals) and become socially acceptable and ethically justifiable.” (p. 624). However, we argue that there is no reason to be worried about this and other examples of enhancement technology *in re.* Ethical issues regarding vaccination merely arise from individual attitudes, non-sufficient scientific communication, and distributive justice concerns, not from an inherently vicious nature. Overall, policymakers and scientists will get some helpful input from studying the general ethical debate on Human Enhancement.

When developing or researching technologies and their ethical implications, one should always ask whether that technology might be a form of Human Enhancement and, if so, how might ethical discomfort be grounded due to that status. This is especially of concern when the target audience of that technology is vast. Scholars and policymakers are well-advised to examine the ethical debate surrounding so many ground-breaking technologies such as smartphones, psychopharmaceuticals or emerging biotechnologies from an explicit Human Enhancement point of view. This also affects medical professionals as they often act as gatekeepers who are expected to regulate the access to various enhancements and respond to the demand of enhancements in different contexts [66, 67]. Empirically showing possible connections between the specific opposition arguments and the general Human Enhancement debate may enhance our understanding of why people are afraid of or affirm certain types of technology by simultaneously sharpening our ethical stand on this manifold phenomenon. However, this requires large, multinational samples and a combination of quantitative and qualitative data.

Examining vaccination against SARS-CoV-2 as a practical example of Human Enhancement shows the difficulties in defining the latter kinds of technologies. However, it provides an excellent example for the debate as to whether or not we should pursue these technologies. Moreover, this example demonstrates the need to take a step back and consider not only the technology in its own right but also its potential social and political impact [23]. Note that we are not arguing for a general laissez-faire approach to Human Enhancement, just because one example of this technology seems to be relatively morally unencumbered. Instead, we are calling for a case-by-case examination of enhancement technologies. Indeed, some technologies need regulation and an intense ethical debate about their impacts. However, the formal status of vaccination against SARS-CoV-2 as Human Enhancement provides no closing argument here. As Daniels [11] has noted, the status as enhancement or treatment does not allow for any definitive conclusion on whether or not an intervention is morally obligated. In the light of the global health crisis, the administration of an enhancement technology is a promising strategy to restore and improve a pre-COVID state. Thus, it may be not only morally obligatory to get vaccinated but also obligatory to any policymaker to ensure a fair distribution of vaccinations inside and beyond state borders.

## Conclusion

Vaccination against SARS-CoV-2 is a practical example of Human Enhancement. This supports the notion that Human Enhancement is not a phenomenon of a distant, dystopian, or even utopian future but is already widely applied. Therefore, rejecting Human Enhancement per se is unjustified, as it would mean to condemn technologies that were used for hundreds of years. Instead of engaging in an overly alarmist or optimistic debate, Human Enhancement technologies suspected of having negative consequences should be examined on a case-by-case basis, taking various factors into account. There are valuable lessons to be learned for any mass vaccination program by comparing the concerns raised with the broader Human Enhancement debate.

## Data Availability

The dataset supporting the conclusions of this article is available in the OSF repository, https://osf.io/spq2a/, DOI 10.17605/OSF.IO/SPQ2A.
